# Overexpression of Adenoviral E1A Sensitizes E1A+Ras-Transformed Cells to the Action of Histone Deacetylase Inhibitors

**Published:** 2018

**Authors:** M. V. Igotti, S. B. Svetlikova, V. A. Pospelov

**Affiliations:** Institute of Cytology, Russian Academy of Sciences, Tikhoretsky Ave., 4, St-Petersburg, 194064, Russia

**Keywords:** apoptosis, histone deacetylase inhibitors, E1A and cHa-ras oncogenes, transformed cells

## Abstract

The adenoviral E1A protein induces cell proliferation, transformation, and tumor formation in rodents, on the one hand. On the other hand, E1A expression increases cell sensitivity to a number of cytotoxic agents. Therefore, E1A is a candidate for use as a component of combination therapy for malignant tumors. The highest augmentation in the cytotoxic effect was achieved by a combined use of E1A expression and histone deacetylases (HDAC) inhibitors. However, HDAC inhibitors do not induce apoptosis in cells transformed with E1A and cHa-ras oncogenes. In this study, it was shown that HDAC inhibitors reduce the expression of adenoviral E1A. However, under unregulated E1A overexpression, these cells undergo apoptosis in the presence of HDAC inhibitors. Treatment with a HDAC inhibitor, sodium butyrate (NaBut), was shown to activate the anti-apoptotic factor NF-kB in control cells. However, NaBut was unable to modulate the NF-kB activity in E1A overexpressed cells. Therefore, it is fair to postulate that cells transformed with E1A and cHa-ras oncogenes avoid the apoptosis induced by HDAC inhibitors thanks to a NaBut-dependent decrease in E1A expression.

## INTRODUCTION

The E1A gene of human adenovirus type 5 is an early response gene that is expressed in infected cells and provides the necessary conditions for virus replication [1]. At first, E1A was considered an oncogene due to its ability to immortalize rodent cells and transform them in cooperation with other oncogenes [2, 3]. It was found later that E1A exhibits antitumor activity [4]; it is sometimes considered a tumor suppressor for that reason.

The transforming activity of E1A is determined by its ability to deregulate the cell cycle by binding to and altering the activity of such cellular factors as pRb family proteins [5-7] and the cyclin-dependent kinase inhibitors p21Waf1 [8, 9] and p27Kip1 [10]. E1A also interacts with chromatin remodeling proteins, including histone acetyltransferase (p300/CBP) [11] and histone deacetylases [12]. This interaction changes the transcription of a number of the genes involved in cell cycle regulation. Adenoviral DNA and the E1A protein are found in the lung epithelium cells of patients with a chronic obstructive pulmonary disease [13]. However, as we have already mentioned, E1A possesses an antitumor activity and is the subject of clinical studies [14, 15]. Plenty of experimental data suggest that expression of adenoviral E1A protein increases the sensitivity of mammalian cancer cells to a number of cytotoxic agents used in antitumor therapy, such as etoposide, cisplatin, taxanes, etc. [16-19]. The combined effect of E1A gene therapy and HDIs leads to a more significant increase in the level of cancer cell death, accompanied by a minimal negative impact on normal cells, as compared to taxol or etoposide [19].

Adenoviral E1A promotes apoptotic cell death by modulating the expression of the genes regulating apoptosis [17-19], the activation of p38 MAP kinase [17], and suppression of the anti-apoptotic factor NFκB [20, 21]. E1A also stabilizes p53 via a modification of the ubiquitin proteasome pathway [16, 22]. As a result, the p53 protein level in cells expressing adenoviral E1A protein increases, leading to p53-dependent apoptosis [16].

The level of apoptosis in cells expressing E1A can be reduced by the complementary transforming *ras* oncogene, which activates the anti-apoptotic PI3K/Akt cascade and NF-κB via the stimulation of the Ras/Raf/MEK/ERK kinase cascade [23]. The anti-apoptotic functions of Ras are associated with its ability to stimulate the expression of the antiapoptotic Bcl-2 and Bcl-XL proteins [23]. Thus, the action of the proapoptotic E1A protein and oncogenic Ras is balanced in mouse embryonic fibroblasts stably transformed by the vector encoding cHa-ras and the plasmid encoding the E1A protein of human adenovirus type 5 [24].

Histone deacetylase inhibitors (HDIs) inhibit tumor cell growth, thus causing cell cycle arrest, senescence, or apoptosis, without having a toxic effect on normal cells [25, 26]. Therefore, HDIs are considered to be promising antitumor agents

We have previously shown that HDIs cause cell cycle arrest and senescence of cells transformed by *cHa-Ras* and E1A oncogenes [27-29] but do not induce their death. This feature distinguishes these cells from other tumor cells, where HDIs stimulate apoptotic death [25, 26]. Therefore, we studied the reasons behind the absence of apoptotic death of cells expressing E1A with activated Ras under the action of HDIs. It was found that the ability of cells transformed by E1A and *cHa-ras* to avoid death under the action of HDIs is due to the HDI-dependent decrease in E1A expression and activation of the NF-κB anti-apoptotic factor. Therefore, induction of apoptosis in E1A+Ras-transformed cells by HDIs is possible only under unregulated E1A expression.

## MATERIALS AND METHODS

**Cell lines**

Our studies were performed using mouse embryonic fibroblasts that had been stably transformed with a vector encoding *cHa-ras* and with p1A plasmid that contained nucleotides 1–1634 of the genome of human adenovirus type 5 encoding the E1A protein [16, 24]. Cells were treated with NaBut (4 mM) for 24–72 h.

**Cell distribution according to DNA content**

The distribution of cells by DNA content was studied by flow cytometry. The cells were washed with a PBS solution (0.14 M NaCl, 2.7 mM KCl, 6.5 mM Na_2_ HPO_4_ , 1.5 mM KH_2_ PO_4_ , pH 7.2), permeabilized with saponin at a final concentration of 0.01% for 20 min, and repeatedly washed with the PBS solution to remove saponin. The cells were then incubated with RNase A (100 µg/mL) and propidium iodide (10 µg/mL, 15 min at 37°C) and analyzed on a Coulter Epicks XL flow cytometer (Bechman, USA).

**Cell viability**

Cell viability was measured by MTT assay. The cells were plated in 96-well plates at a density of 2 × 10^3^ cells/well and cultured for 24 h in either the presence or absence of the respective inhibitors. Cell viability was determined spectrophotometrically by assessing their metabolic activity according to their ability to reduce 3-(4,5-dimethylthiazole-2-yl)-2,5-diphenyltetrazolium bromide (MTT) (Sigma) to insoluble purple formazan. The cells were incubated in a MTT solution in PBS at a final concentration of 0.5 mg/mL (1.5 h at 37°C in a CO_2_ incubator). The culture medium was then removed, and the cells were suspended in dimethyl sulfoxide (DMSO). The optical density in each well was determined at a wavelength of 570 nm using a Multiscan-EX plate reader (Labsystems). DMSO was used as a blank control.

**Protein immunoblotting**

The cells were lysed in a buffer containing 1% NP40, 0.5% sodium deoxycholate, 0.1% sodium dodecyl sulfate (SDS), protease, and phosphatase inhibitors. Proteins were separated by electrophoresis, transferred to a PVDF membrane (Millipore), and analyzed using the appropriate specific antibodies. Proteins on the membranes were detected using the enhanced chemiluminescence method (Thermo Sci., USA). Antibodies raised against E1A (M73) proteins (Santa Cruz Biotechnology, Inc., USA), Gapdh (14C10) (Cell Signalling, USA) and pan-Ras proteins (Oncogene Sci., USA) were used.

**Gene transcription analysis**

Cellular RNA was isolated using a Trizol reagent (Invitrogen, USA). The reverse transcription reaction was performed using 2 µg of RNA. The amplification reaction (PCR) was performed in the presence of 100 ng of the corresponding primers to cDNA of the mouse e1a and gapdh genes: 5’-TGTGATGGGTGTGAACCACG-3’/5’-CCAGTGAGCTTCCCGTTCAG-3’. Linear PCR amplification of DNA fragments was performed during 25–35 cycles. The specific reaction product was analyzed by electrophoresis in 2% agarose gel.

**Caspase-3 activity**

Caspase-3 activity was assayed in vitro based on the cleavage of the specific colorimetric substrate Ac-DEVD-pNA (Calbiochem). Cells were lysed for 20 min at +4°C in a buffer containing 50 mM Tris-HCl, pH 7.5; 120 mM NaCl; 1 mM EDTA; 1% NP-40, and protease inhibitors. Caspase activity was determined in 96-well plates in 40 µL of lysates mixed with 160 µL of a reaction buffer (20% glycerol; 0.5 mM EDTA; 5 mM DTT; 100 mM HEPES, pH 7.5) containing Ac-DEVDpNA substrate. The efficiency of Ac-DEVD-pNA cleavage was determined spectrophotometrically based on the accumulation of *n*-nitroanilide at a wavelength of 405 nm using a Multiscan-EX spectrophotometer (Labsystems).

**Temporary transfection and analysis of luciferase activity**

Cells were transfected using the Lipofectamine-2000 reagent (Invitrogen), according to the manufacturer’s protocol. A luciferase reporter vector carrying three copies of NF-κB-binding sequences (3 × κB-luc) was used for transfection. Renilla luciferase expression was used as an internal control. Cells were treated with 4 mM NaBut 24 h post-transfection and then processed according to the manufacturer’s instructions for measuring luciferase activity after 48 h. Luciferase activity was determined using a TD-20/20 luminometer (Turner Designs). Each experiment was repeated at least 3 times.

## RESULTS

**Histone deacetylase inhibitor sodium butyrate inhibits the expression of adenoviral E1A**

**Fig. 1:**
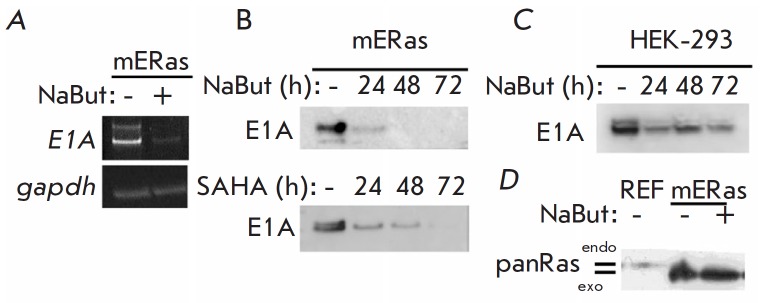
Sodium butyrate downregulates E1A expression in cells expressing E1A. *A *– RT-PCR analysis of *E1A *transcription in mERas cells: either untreated (-) or treated (+) with 4 mM NaBut for 16 h; *B *– immunoblotting of proteins from mERas cells, either untreated (-) or treated with 4 mM NaBut (upper panel) or 2.5 μM SAHA (lower panel) for 24–72 h, with antibodies raised against E1A of human adenovirus type 5 (E1A5Ad); *C *– immunoblotting of proteins from HEK-293 cells with antibodies raised against E1A5Ad; *D *– immunoblotting of proteins from mERas cells with antibodies raised against pan-Ras

In order to clarify the reasons for the absence of the expected cytotoxic effect of HDIs in E1A-expressing cells, we analyzed the effect of a HDI, sodium butyrate (NaBut), on the expression of transforming oncogenes. The data presented in *Fig. 1* show that E1A expression is reduced in the presence of NaBut. In mouse embryo fibroblasts transformed with the E1A and *cHa-Ras* oncogenes (mERas line), transcription of the *e1a* gene (*Fig. 1A*) and the level of E1A protein (*Fig. 1B*) decreased as soon as during the first hours of exposure to NaBut. Is this effect specific to the particular cell line and HDIs employed? In order to answer this question, we analyzed the E1A expression in the transformed human cell lines and used alternative HDIs. We found that valproic acid (VA), trichostatin A (TSA) (data not shown), and vorinostat also reduced the amount of E1A in mERas transformants (*Fig. 1B, lower panel*). The results shown in *Fig. 1C* demonstrate that the decrease in E1A expression under the action of HDIs is not specific only to the mERas line. Immunoblotting demonstrated that the amount of E1A protein in the transformed human renal epithelial HEK-293 cells decreased in the presence of NaBut. The results of immunoblotting showed that the expression of the Ras protein did not change in the presence of NaBut (*Fig. 1D*). Thus, HDIs were found to suppress the expression of adenoviral E1A, whereas Ras expression was not modulated by HDIs.

The detected decrease in the E1A protein level in the presence of HDIs can shift the equilibrium between the activities of transforming proteins in mERas cells. Meanwhile, the action of oncogenic Ras becomes dominant. We considered that the low level of apoptosis in the E1A+Ras transformed cells treated with HDIs was associated with the HDIs-mediated decrease in pro-apoptotic E1A protein expression and activation of the anti-apoptotic Ras/Akt/NF-κB cascade.

**Production of a E1A+Ras-transformed cell line with E1A expression unregulated by HDIs**

The mERas cell line was obtained using a p1A plasmid carrying the nucleotides 1–1634 of human adenovirus type 5 encoding the E1A protein [16, 24]. In these cells, the native promoter regulates the expression of the *e1a *gene. In order to test the hypothesis that reduction in E1A expression is required to reduce the HDIs-induced apoptosis, we produced a MER-E1A cell line based on mERas cells. The MER-E1A cell line additionally expressed the E1A 12S protein under the control of an unregulated cytomegalovirus (CMV) promoter. Regulation and activity of CMV and Ad5 viral promoters differ significantly: so, they can be used in target cells for different purposes. The high-activity CMV promoter is convenient for efficient transgene expression.

**Fig. 2:**
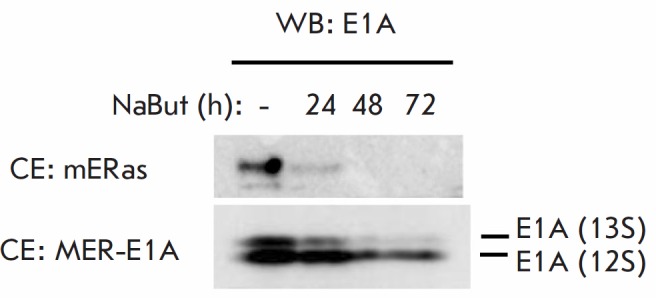
Immunoblotting of proteins from mERas (upper panel) and MER-E1A (lower panel) cells treated with 4 mM NaBut for 24–72 h, with antibodies raised against E1A5Ad

*Figure 2* shows the immunoblotting results that demonstrate how NaBut affects the expression of the E1A protein in the original mERas cell line (upper panel) and in the new MER-E1A cell line with constitutive E1A expression under the control of the CMV promoter (lower panel). In the control mERas cells, E1A expression decreased to almost zero already during the first hours of exposure to NaBut and remained at a low level throughout the entire study (up to 72 h). However, in MER-E1A cells, the E1A 12S protein was expressed at a high level independently of NaBut.

Thus, we obtained a line of transformed rodent cells expressing the adenoviral E1A gene under the control of the CMV promoter in which E1A expression did not decrease in the presence of HDIs.

**Sodium butyrate induces apoptosis only in E1A+Ras-transformed cells where the amount of E1A does not decrease in the presence of HDIs**

**Fig. 3:**
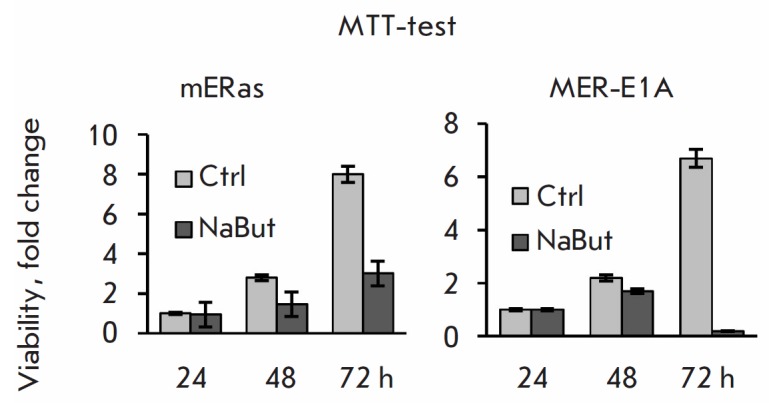
Sodium butyrate suppresses the viability of mERas and MER-1A cells to different extents. Control mERas and MER-E1A cells stably expressing E1A were treated with NaBut for 24–72 h, and their viability was determined by MTT assay. Changes in viability (fold) were assessed with respect to the viability of untreated cells 24 h after plating

Next, we compared the effect of HDIs on the proliferation of E1A+Ras-transformed cells where E1A is expressed under the control of an intrinsic promoter, and in cells where the CMV promoter regulates E1A expression. We evaluated the effect of NaBut on cell viability depending on E1A expression. The control mERas cells and MER-E1A cells with unregulated E1A expression were treated with NaBut for 24–72 h; their viability was determined using the MTT assay. *Figure 3A* demonstrates that the viability of control mERas cells treated with NaBut decreased more than that of the untreated ones. However, the amount of formazan, which characterizes cell viability, rose with an increase in the duration of exposure of mERas cells to NaBut. The increased amount of formazan attests to the fact that the cells did not divide but remained alive. Meanwhile, the number of viable MER-E1A cells decreased below the baseline, indicating cell death.

**Fig. 4:**
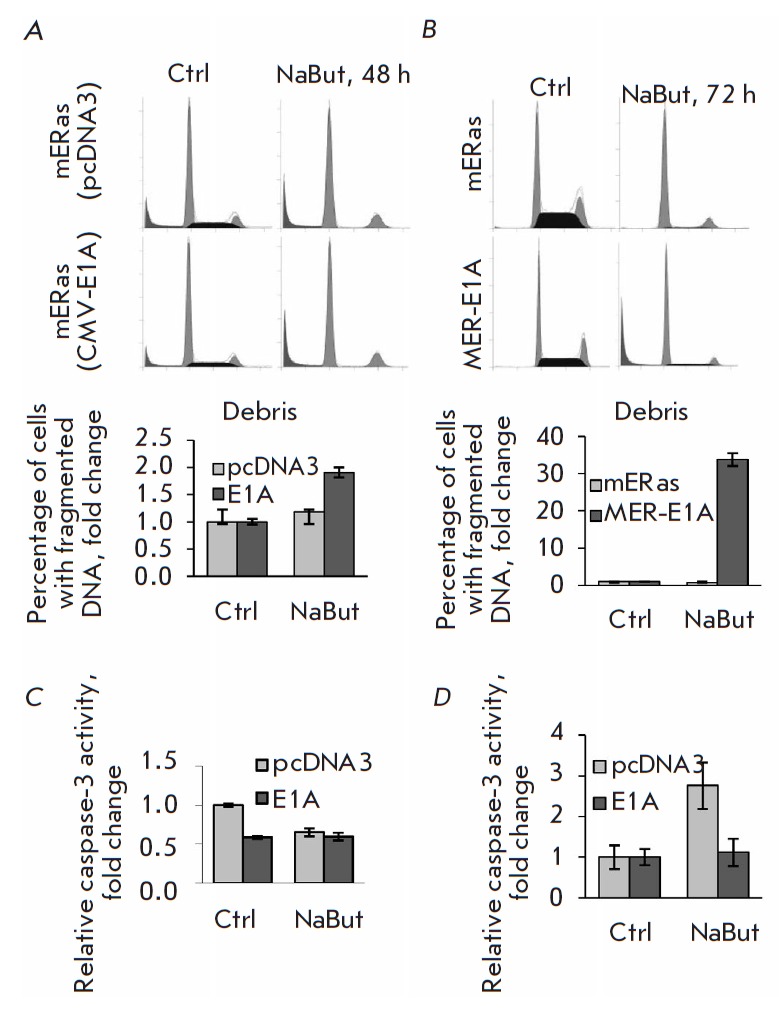
Adenoviral E1A alters the effect of NaBut on transformed cells. *A *– FACS analysis of mERas cell distribution according to DNA content. The mERas cells were transfected with the control vector pcDNA3 (upper panel) or expression vector CMV-E1A (lower panel); after 24 h, the transfected cells were treated with NaBut for 48 h; *B *– distribution of stable clones according to DNA content. The mERas control cells or MER-1A cells stably expressing E1A were treated with NaBut for 72 h; *C *– the relative activity of caspase-3 in cells transfected with pcDNA3 (light gray bars) or CMV-E1A (dark bars), untreated (Ctrl) or treated with 4 mM NaBut for 24 h; *D *– the relative activity of luciferase transcribed from a NF-κB-regulated promoter. The mERas cells were co-transfected with a reporter 3*kB-luc vector and empty pcDNA3 vector (light gray bars) or with a CMV-E1A expression vector (dark bars) and treated with NaBut 24 h post-transfection for 24 h

In order to test the hypothesis about the induction of death of cells with E1A expression not regulated by NaBut, we analyzed the distribution of cells by DNA content using a flow cytometer. Cell distribution after transient transfection is shown in *Fig. 4A*. One can see that NaBut did not increase the sub-diploid peak in cells transfected with the control empty pcDNA3 vector (*Fig. 4A*, upper panel). At the same time, the percentage of cells with a sub-diploid DNA content in cells transfected with CMV-E1A increased twofold already 48 h after the exposure to NaBut (*Fig. 4A*, bottom panel). The findings demonstrate that there is a significant difference in cell response to HDIs depending on how HDIs modulate E1A expression.

The corresponding results were obtained in stable clones with E1A overexpression under the control of the CMV promoter (MER-E1A). In the control mERas cells, NaBut did not increase the sub-diploid peak in the distribution histogram of DNA content, which is characteristic of dying cells, even after 72 h (*Fig. 4B*). Meanwhile, 35% of MER-E1A cells contained fragmented DNA 72 h after the exposure to NaBut.

Hence, it was shown that NaBut induced the death of only those Ras-transformed cells in which expression of E1A did not decrease in the presence of NaBut.

We analyzed the activity of the caspase-3 mediating the transduction of the apoptotic signal. For this purpose, cells transfected with pcDNA3 or CMV-E1A were either left untreated or treated with NaBut for 24 h; the *in vitro *activity of caspase-3 in cell lysates was subsequently determined. NaBut reduced caspase-3 activity in control cells transfected with pcDNA3, identically to the case in the initial mERas cells [30]. Meanwhile, NaBut did not reduce caspase-3 activity in cells transfected with CMV-E1A (*Fig. 4C*). The differences in the regulation of caspase-3 activity by HDI depending on modulation of the E1A expression are consistent with our data demonstrating differences in the proliferative response of these cells to HDI.

**NaBut does not increase NF-κB activity in cells with unregulated E1A expression**

It was shown earlier that HDIs activate the anti-apoptotic factor NF-κB in cells transformed with E1A and *cHa-ras *[30]. This activation allowed transformants to avoid apoptosis when exposed to HDIs. Therefore, we compared the effect of HDIs on NF-κB activity in cells with regulated and unregulated E1A expression. For comparison, the initial mERas cells were co-transfected with a 3×κB-luc vector containing the luciferase gene under the control of a promoter regulated by NF-κB and either the CMV-E1A expression vector or empty pcDNA3 vector as a control. Twenty-four hours post-transfection, the cells were either left untreated or treated with NaBut for 24 h. Luciferase activity in lysates was measured. The NF-κB-dependent transcription in the control (pcDNA3) cells increased threefold in the presence of NaBut, whereas NF-κB activity in cells with unregulated high E1A expression (CMV-E1A) remained unchanged (*Fig. 4D*). We found that unregulated high expression of adenoviral E1A from the CMV promoter prevented HDI-dependent activation of the anti-apoptotic factor NF-κB. Therefore, inhibition of NF-κB activity by adenoviral E1A is one of the reasons for the induction of apoptosis by HDIs in these cells.

**Cells with unregulated E1A expression do not accumulate senescence marker SA-β-Gal in the presence of NaBut**

**Fig. 5:**
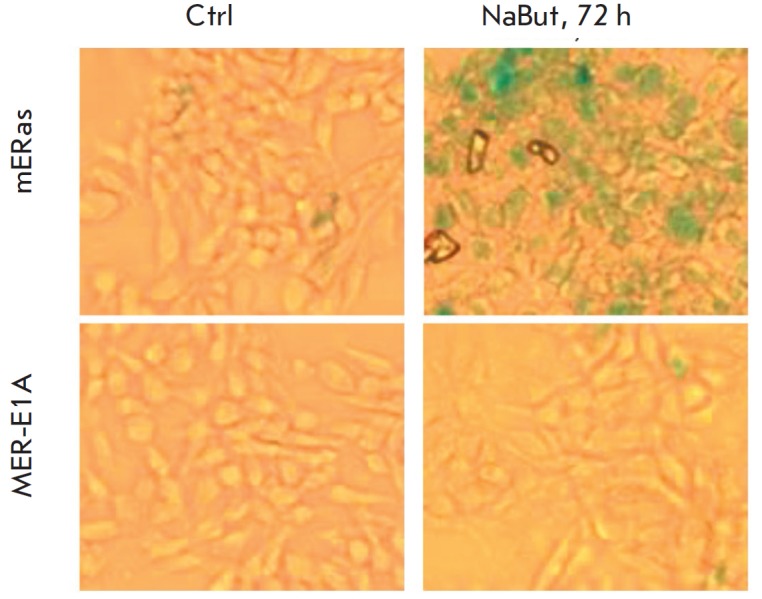
Sodium butyrate failed to induce senescence in cells with unregulated E1A expression. SA-β Gal staining. The mERas and MER-E1A cells were treated with NaBut for 72 h; the cells were then fixed and stained for SA-βGal

Cellular senescence and apoptosis are the alternative anti-proliferative programs induced by cytotoxic and stress factors. It was previously shown that HDIs induce senescence of cells transformed with *cHa-Ras *and E1A oncogenes [27-29]. The senescence program in these cells is presumably initiated due to the fact that HDIs downregulate E1A expression: so, the cellular senescence program induced by activated Ras starts to predominate [31]. In order to check the assumption that cellular senescence is not induced in E1A+Ras-transformed cells where E1A expression is not suppressed by HDIs, we analyzed the expression of the cellular senescence marker SA-β-Gal. The optical microscopy data (*Fig. 5*) show that there is SA-β-Gal in almost all control mERas transformants after treatment with NaBut for 72 h, thus indicating that cellular senescence was induced. In MER-E1A cells, the SA-β-Gal marker had not accumulated. After exposure to NaBut for 72 h, very few MER-E1A cells remained attached to the slides for further SA-β-Gal staining; most cells died and were floating. Hence, it is fair to conclude that NaBut induces cellular senescence in cells transformed with *E1A* and *Ras* if E1A expression decreases in the presence of NaBut. Meanwhile, the transformants with constitutive E1A expression die rather than senesce.

## DISCUSSION

In cooperation with activated *Ras *or other oncogenes, adenoviral E1A immortalizes and transforms primary rodent cells [2, 3]. In this regard, E1A was earlier considered to be an oncoprotein, although it was not associated with any oncogenic activity. Later, E1A was found to exhibit antitumor activity [2, 17-19]. Overexpression of E1A causes arrest of proliferation and apoptosis of human tumor cells *in vitro *[4, 16]. Moreover, apoptosis plays a key role in the antitumor activity of E1A. A number of preclinical studies demonstrated that liposomal or adenoviral delivery of the E1A gene inhibits tumor growth and metastasis development in animals [17, 32]. Clinical trials of gene monotherapy and combination therapy with E1A for cancers of different localizations demonstrated that this approach is justified [14, 33, 34]. The efforts of many scientists have recently been focused on whether or not therapy using oncolytic viruses based on human adenovirus type 5 (Ad5) can be employed [35]. According to the ClinicalTrials.gov website of the National Institutes of Health, more than 180 clinical trials using adenoviruses in a particular form have already been conducted. The gene encoding E1A is the main target for the production of oncolytic adenoviruses with limited replicative ability. This choice is determined by the role played by this protein in the initiation of cell division in a resting cell via sequestration of the tumor suppressor pRb. Taking into account the potential significance of adenoviruses and adenoviral E1A in antitumor therapy, comprehensive research into the functioning and regulation of E1A, which leads to cell sensitization to cytostatics, is a relevant issue in molecular biology.

Many studies have demonstrated that combined use of HDIs and adenoviral E1A in tumor cells increases the cytotoxic effect [19, 36, 37]. HDIs did not cause the death of cells transformed with the *cHa-ras *and *E1A *oncogenes used in this study. However, sodium butyrate induced apoptosis in these cells when adenoviral *E1A *was expressed under the control of a HDI-unregulated promoter. In studies reporting that combined use of HDIs and E1A enhanced the cytotoxic effect, *E1A *expression was controlled not by a native promoter, but by the cytomegalovirus (CMV) promoter or by the catalytic subunit of telomerase (TERT) [19, 39-40]. The activity of these promoters was not suppressed by HDIs but stimulated by them [19, 38]. Consequently, our results are in line with the data on the efficiency of a combined use of HDIs and E1A for eliminating malignantly transformed cells under increased unregulated E1A expression. Our results are of higher priority as we have demonstrated that HDIs can suppress E1A expression on several levels. First, transcription of the E1A gene was reduced in the presence of NaBut (*Fig. 1A*). The regulation of E1A expression is currently understudied. No data on the role of acetyltransferases or deacetylases in the regulation of E1A transcription are available. Inhibition of histone deacetylases activates gene transcription via a relaxation of the chromatin structure. On the other hand, HDIs can inhibit or activate transcription by changing the acetylation level of transcription factors [41]. Hence, the demonstrated inhibition of E1A is probably mediated by a modulation of the activity of the transcription factors involved in the regulation of E1A expression by histone deacetylases. The enhancer region of the E1A promoter contains two binding sites for E2F transcription factors, along with other regulatory elements [42]. The absence of these sites completely suppresses E1A expression, thus indicating that the E2F-binding regions play an exceptionally important role in the regulation of *E1A *transcription. Earlier, we showed that NaBut suppresses the *trans*-activating ability of the E2F factor [28, 43]. Therefore, it is fair to assume that the observed decrease in E1A expression is partially due to NaBut-dependent inhibition of the E2F factor. The activity of other viral promoters frequently used in genetic engineering (cytomegalovirus and polyomavirus (SV40)) is known to be stimulated by HDIs [38]. Although sharing a number of similar features, viral promoters differ significantly in terms of the mechanism of regulation of their activity. Thus, the CMV promoter is positively regulated by the E1A protein [44], whereas the E1A protein represses the native promoter of the E1A gene and HIV-LTR promoter [45, 46]. Therefore, it can be assumed that regulation of the activity of viral promoters by deacetylase inhibitors is also not universal.

Second, our data suggest that HDIs reduce the content of the E1A protein both in mERas cells and in a transformed human embryonic kidney (HEK) 293 cell line (*Fig. 1B*). Moreover, the content of the E1A protein decreases more intensively than *E1A* transcription does. This finding indicates that deacetylase inhibitors modulate the stability of the E1A protein. Like many cellular proteins, virus-encoded proteins also act as substrates for acetyltransferases and deacetylases. The E1A protein is able to bind to p300/ CBP and can be acetylated by p300 and PCAF [47]. Acetylation alters the nature of the interaction between E1A and partner proteins [48] and determines its intracellular localization [47]. Thus, E1A acetylation prevents nuclear import and, accordingly, leads to E1A accumulation in the cytoplasm. However, inhibition of deacetylases by sodium butyrate in HEK-293 cells expressing E1A did not increase the amount of acetylated E1A and, consequently, did not cause accumulation of E1A in the cytoplasm [47]. These data suggest that E1A undergoes rapid degradation that follows protein acetylation. The E1A degradation can occur in proteasomes [48]. It was also shown that early-region 1A proteins of adenovirus type 2 and 12 (Ad2 and Ad12 E1A) were cleaved by caspases-3 and caspases-7 during induced apoptosis in human and mouse cells transformed by adenovirus [49]. The aforementioned data suggest that enhanced acetylation of the E1A protein induced by HDIs may be one of the factors responsible for E1A degradation.

Comparison of the responses of transformed cells with regulated and unregulated E1A expression to HDIs showed that apoptosis was induced only in cells with an increased unregulated E1A expression. In the control mERas cells with a reduced content of E1A, the cell senescence program was initiated (*Fig. 5*). We have shown that avoidance of apoptotic death by control mERas cells is associated with the downregulated expression of the pro-apoptotic E1A protein and activation of the anti-apoptotic NF-κB cascade. Meanwhile, the oncogenic Ras inducing senescence starts to play a predominant role. In turn, induction of apoptotic death in the presence of NaBut in cells with E1A overexpression is associated with a suppressed and unregulated activity of the anti-apoptotic NF-κB complex. Data on the repression of NF-κB activity by E1A oncoprotein have been reported [18, 20, 21]. Thus, E1A competitively binds and inactivates protein kinase A, which is expected to phosphorylate NF-κB and thus activate it [21]. E1A also suppresses IKK activity, thus reducing the degradation of IκB, the inhibitor regulating the NF-κB function [20]. Therefore, taking into account the aforementioned data and the findings that demonstrate that E1A content and activation of NF-κB decrease in a time-synchronized manner [30], it is fair to say that HDIs affect the NF-κB activity in cells transformed with E1A+Ras by modulating E1A expression.

## CONCLUSIONS

Expression of adenoviral E1A increases the sensitivity of tumor cells to apoptosis-inducing agents [18]. Therefore, E1A is of great interest as a potential component of combination tumor therapy. The combined use of E1A and HDIs enhances the cytotoxic effect in many cancer cells, while having a minimal negative effect on normal cells [19]. However, HDIs do not induce apoptosis in a cell line transformed with *cHa-Ras *and E1A oncogenes in which E1A is expressed under the control of a native viral promoter. In the present study, we have shown that HDIs suppress the expression of adenoviral E1A. Apoptotic death of E1A/Ras-transformed cells can be induced by HDIs if E1A is expressed at a high unregulated level. In other words, the avoidance of apoptotic death by *Ras*-transformed cells expressing E1A is associated with downregulation of E1A expression in the presence of HDIs. In turn, the forced HDI-independent expression of E1A paves the way for apoptosis induction.

## Abbreviations

HDAC: histone deacetylase
HDI: histone deacetylase inhibitor
mERas: mouse embryo fibroblasts transformed with E1A and cHa-ras oncogenes
NaBut: sodium butyrate
